# Quantification by SIFT-MS of volatile compounds produced by the action of sodium hypochlorite on a model system of infected root canal content

**DOI:** 10.1371/journal.pone.0198649

**Published:** 2018-09-10

**Authors:** Konstantinos Ioannidis, Sadia Niazi, Sanjukta Deb, Francesco Mannocci, David Smith, Claire Turner

**Affiliations:** 1 Division of Tissue Engineering and Biophotonics, Dental Institute, King’s College, London, United Kingdom; 2 Department of Restorative Dentistry, Dental Institute, King’s College, London, United Kingdom; 3 Trans Spectra Limited, Newcastle-under-Lyme, United Kingdom; 4 School of Life, Health and Chemical Sciences, The Open University, Walton Hall, Milton Keynes, United Kingdom; National Taiwan University, School of Dentistry, TAIWAN

## Abstract

Root canal irrigation with sodium hypochlorite (NaOCl) is an indispensable part of the chemomechanical preparation of infected root canals in Endodontology. However, there is limited information on the emergence of toxic or hazardous volatile compounds (VOCs) from the interaction of NaOCl with the infected content of tooth biomaterials. The aim of this study was to assess the formation of VOCs and disinfection by-products (DBPs) following the interaction of NaOCl 2.5% v/v with a model system of different sources of natural organic matter (NOM) present in infected root canals, including dentine powder, planktonic multi-microbial suspensions (*Propionibacterium acnes*, *Staphylococcus epidermidis*, *Actinomyces radicidentis*, *Streptococcus mitis and Enterococcus faecalis strain OMGS3202*), bovine serum albumin 4%w/v and their combination. NaOCl was obtained from a stock solution with iodometric titration. Ultrapure water served as negative control. Samples were stirred at 37°C in aerobic and anaerobic conditions for 30min to approximate a clinically realistic time. Centrifugation was performed and the supernatants were collected and stored at -80^0^ C until analysis. The reaction products were analysed in real time by selected ion flow tube mass spectrometry (SIFT-MS) in triplicates. SIFT-MS analysis showed that the released VOCs included chlorinated hydrocarbons, particularly chloroform, together with unexpected higher levels of some nitrogenous compounds, especially acetonitrile. No difference was observed between aerobic and anaerobic conditions. The chemical interaction of NaOCl with NOM resulted in the formation of toxic chlorinated VOCs and DBPs. SIFT-MS analysis proved to be an effective analytical method. The risks from the rise of toxic compounds require further consideration in dentistry.

## Introduction

Sodium hypochlorite (NaOCl) is one of the of the most widely practised public health components of disinfection of drinking water, sewage-water plants, water supply and distribution systems, swimming pools and industrial applications with the aim of preventing the spread of infection and contamination [1, 2]. Chlorination can kill the majority of bacteria, viruses and parasites responsible for waterborne diseases [3], as a strategy to meet the drinking water quality standards and disinfect waste water [4, 5]. Despite the importance of water disinfection, potentially harmful halogenated disinfection by-products (DBPs) can result from the reaction of chlorine or hypochlorite (which forms hypochlorous acid in water) with natural organic matter (NOM) in water, having negative health effects [6–8].

Several categories of DBPs are currently under investigation. To date, more than 300 species have been identified [9]. Historically, the first and most widely studied group of DBPs are trihalomethanes (THMs) [10] followed by haloacetic acids (HAAs) [11, 12]. Other prevalent categories of DBPs are haloaldehydes, haloketones, haloacetonitriles, chloropicrin and chlorophenols [13]. The presence of DBPs in the domestic water supply results in widespread exposure via inhalation and direct ingestion through water consumption, and dermal absorption through bathing, showering, and swimming [14–17]. Daily exposure to chlorinated water may be dangerous to human health because of the carcinogenic and mutagenic properties of these DBP compounds [18, 19]. A 30-year summary of the potential cytotoxic and mutagenic effects of DBPs, which provide data and risk factors with regard to occurrence, genotoxicity, carcinogenicity and health concerns, was produced by Richardson *et al*. [9].

The use of NaOCl has been universally adopted and extensively used in endodontics as the main irrigant for the disinfection of infected root canals. This is due to its antimicrobial properties [20], its ability to disintegrate and solubilize organic tissue [21] and to denature toxins [22]. In addition, it is inexpensive, has a long shelf life and is readily available [23]. The proposed concentration for clinical use in dentistry varies from 1.00% to 5.25% v/v in an attempt to balance its beneficial action whilst considering its negative effects on dentine’s biomechanical properties, cellular cytotoxicity and caustic effects [24]. Clinical studies have indicated both low and high concentrations to be equally effective in reducing bacteria from the root canal system, however a general consensus exists that 2.5% v/v is the most commonly used concentration clinically and is considered acceptable both in terms of NaOCl solubilising and antimicrobial capacity in Endodontics [25, 26].

NaOCl is a proteolytic agent which promotes a wide range of chemical interactions with the multivariable content of the root canal system. During root canal chemomechanical preparation, NaOCl interacts with radicular and coronal dentine, leading to concentration and time dependent denaturation of the collagenous organic matrix and alterations of the structural integrity of mineralised dentine [27, 28]. Whilst instrumentation progresses and irrigation depth increases, NaOCl comes in contact with the infected content of the root canal system. Microbial biofilms and associated endotoxins and metabolic products, vital or necrotized cellular structures, blood and plasma, tissue exudates, vital healthy, inflamed or necrotic pulp tissue and overall organic and inorganic matter and debris formed after mechanical instrumentation comprise the multivariable content [29]. Thus, a constant reservoir of natural organic matter (NOM) becomes available during the chemomechanical preparation of the root canal system.

One of the side effects of the chemical interactions of NaOCl, which has received relatively little attention in the literature, is the formation of harmful chlorinated DBPs and additional chlorinated volatile organic compounds (VOCs). The study by Varise *et al*. [30] was the first to report the detection of several volatile organochlorine compounds, following the 15-min interaction of NaOCl with bovine dentine powder and pulp tissue fragments, using gas chromatography-mass spectrometry (GC-MS). The identification of the occurring reactions, the production of DBPs and other organochlorine compounds requires mass- spectrometry based instrumentation. GC-MS is a powerful method for the identification of compounds. However, it is time consuming and often requires pre-concentration of gaseous or volatile components of samples.

The emergence of toxic chlorinated DBPs and VOCs from the interaction of NaOCl with infected root canal content requires further examination due to the potential hazardous drawbacks during root canal irrigation. Selected ion flow tube mass spectrometry (SIFT-MS) is a rapid and direct analytical tool for analysis of VOCs and DBPs compared to GC-MS [31, 32]. This technique is sensitive, fast and quantitative. The use of SIFT-MS may prove to be a valuable method to study the potential reactions of NaOCl with organic material in a laboratory model of infected tooth biomaterial.

The aim of this study was to screen and quantify *ex vivo*, the formation of chlorinated DBPs and VOCs resulting from the short-term interaction of NaOCl 2.5% v/v with human dentine, endodontic pathogens, serum albumin and their combinations, using SIFT-MS and determine the suitability for this analysis. The null hypothesis was that the interaction of NaOCl with these components of infected root canal does not result in the formation of chlorinated DBPs and VOCs.

## Materials and methods

### Dentine preparation

Fifteen freshly extracted intact and fully developed human impacted and semi-impacted mandibular third molars that were free of cracks, fractures, caries, abrasions and discolouration were collected. Informed and written consent was obtained by medically-fit patients, who were referred by their dentists to have their teeth extracted in dental hospital premises. All procedures were approved and conducted in accordance with the protocol outlined by the Research Ethical Committee (Wales REC 4, 14/WA/1004, UK). Access cavities were performed to remove pulpal debris with stainless steel hand files and distilled water. The enamel and cementum layers were removed with high speed diamond conical burs (Ash HiDi Friction Grip Taper 554-Medium Grit, Dentsply, Weybridge, UK). The remaining dentine bulk was then reduced to a powder state by using low speed round burs (Steel Round Finishing Bur size E, Dentsply, Weybridge, UK).

Dentine powder particle size distribution was measured with a laser diffraction particle-size analyser (CILAS 1180, Orleans, France), which could analyse particles within the range 0.04–2500 μm. Five samples of dentine powder (10 mg) were randomly selected, inserted into a water tank and ultra-sonicated for 30 s. Particle size distribution was expressed by the obtained values from the cumulative distributions with measures of central tendency [median diameter (d50)] and distribution width (relative span = d90-d10 / d50) [33]. D50 values varied from 17.66 μm to 31.03 μm and relative span values varied from 1.10 to 2.62. The total dentine powder sample was equally divided into experimental and control groups, each one containing 50 mg, sterilised in an autoclave and kept at -80 ^0^C until further use, to prevent degradation of collagen components.

### Characterisation of root canal irrigant NaOCl 2.5% v/v

The available chlorine content of a 10–15% v/v stock solution of NaOCl (Sigma Aldrich, Gillingham, UK) was verified with a standard iodine/thiosulfate method [34]. The chemical reactants used were: (i) Sodium thiosulphate solution (0.2M) which was prepared from 50 g sodium thiosulphate pentahydrate crystals (99.8% purity, Alfa Aesar, Heysham, UK) dissolved in 1L HPLC water (Chromasolv, Sigma Aldrich, Gillingham, UK), and 25 mg of sodium carbonate (VWR, UK) were added to adjust the pH of the solution to alkaline levels, (ii) potassium iodate (0.05M) (Sigma Aldrich, Gillingham, UK) and potassium iodide (0.24M) (Alfa Aesar, Heysham, UK), (iii) sulphuric acid (3M) (Fisher Scientific, Loughborough, UK) and (iv) potato starch (2% w/v) (VWR, Lutterworth, UK).

Titrations (n = 3) of sodium thiosulphate consumption were carried out after the standardisation of the titrating agent with potassium iodate (0.05M) as a primary standard at 0.22M. The concentration of NaOCl stock solution was 1.474M. Stock NaOCl solution was diluted with HPLC water to obtain 2.5% v/v NaOCl (0.34M). The NaOCl solutions were stored in air-tight dark containers at 5^o^ C until further use.

### Development of planktonic multi-microbial colonies

*Propionibacterium acnes*, *Staphylococcus epidermidis*, *Actinomyces radicidentis* and *Streptococcus mitis*, recovered in previous study as predominant taxa from the root canals of teeth with refractory endodontic infections, were selected [35]. *Enterococcus faecalis* strain OMGS 3202 was also included [36]. To establish the microbial growth, the strains were cultured anaerobically at 37°C for one week on Fastidious Anaerobe Agar (FAA, Lab M, Heywood, UK) supplemented with 5% defibrinated horse blood. Individual starter cultures of each species were collected with inoculation loops (Cole-Palmer, UK), added to phosphate buffered saline (PBS) and incubated anaerobically at 37°C for 3 h in anaerobic workstation (MACS-MG-1000, Don Whitley Scientific Ltd, UK). The absorbance was adjusted with PBS to 0.5 at 540 nm to obtain 10^7^ cells mL^-1^ (Labsystems iEMS Reader MF, Basingstoke, UK).

### Interaction of NaOCl in ultrapure water with different sources of NOM

To study the interaction of NaOCl with combined sources of NOM and their effect on the formation of any chlorinated DBPs and other VOCs, four experimental groups containing aqueous suspensions of NaOCl 2.5% v/v and one control group containing ultrapure water and not NaOCl were developed (Table 1). The total volume for each group was 10 mL, equally divided into four aliquots (2.5 mL), according to the type and combination of NOM sources (Table 1). Dentine powder (50 mg) was suspended in 2.5mL ultrapure water (Sigma Aldrich, UK). Multi-microbial liquid suspensions of 2.5 mL volume were acquired after the addition of 0.5 mL aliquots of each of the five planktonic microbial species. Bovine serum albumin (BSA) solution (4% w/v) was obtained following the addition of BSA powder (A1933, Sigma Aldrich, UK) into ultrapure water (Sigma Aldrich, UK). All samples were injected into 20 mL-universal tubes and stirred at 37 ^0^C under both aerobic and anaerobic conditions for 30 min to approximate a clinically realistic time of root canal irrigation procedures and ensure homogeneous contact between the reactants [37]. After this period, all samples were centrifuged (2 min at 200 rpm) and the supernatants were collected and stored at -80 ^0^C until analysis. The preparation of the aliquots and their associated samples as well as the experimental procedures were conducted in triplicates.

**Table 1:** 

TOTAL VOLUME 10 mL	EXPERIMENTAL GROUPS				CONTROL GROUP
	Group 1	Group 2	Group 3	Group 4	
2.5 mL	NaOCl 2.5%	NaOCl 2.5%	NaOCl 2.5%	NaOCl 2.5%	Ultrapure water
2.5 mL	Dentine powder (50 mg)	Planktonic multi-microbial suspensions	Dentine powder (50 mg)	Dentine powder (50 mg)	Dentine powder (50 mg)
2.5 mL	Ultrapure water	Ultrapure water	Planktonic multi-microbial suspensions	Planktonic multi-microbial suspensions	Planktonic multi-microbial suspensions
2.5 mL	Ultrapure water	Ultrapure water	Ultrapure water	Bovine serum albumin 4%	Bovine serum albumin 4%

Group allocation and interaction of different sources of NOM with NaOCl (Groups 1–4) and ultrapure water (control group). Each sample was studied under aerobic and anaerobic conditions.

### SIFT-MS analysis

The SIFT-MS technique has been extensively described elsewhere [31, 32]; however, a brief explanation is warranted here. In SIFT-MS, a mixture of reagent ions (H_3_O^+^, NO^+^ and O_2_^+^) are generated in a microwave discharge. Each of these reagent ions can be selected by a quadrupole mass filter and separately injected into a fast-flowing helium carrier gas in a flow tube. The sample gas to be analysed naturally flows into the helium at a controlled rate via a calibrated capillary by virtue of the atmospheric pressure of the sample gas and the much lower pressure of the helium (typically 1 mbar). The chosen reagent ion then reacts with the trace components in the sample (to the exclusion of the major air components) to generate product (analyte) ions. The reagent ions and analyte ions are mass analysed by a quadrupole mass spectrometer and counted by a detector. Thus, the characteristic analyte ions identify the neutral trace components present in the sample and their count rates provide their concentrations in real time.

For analysis, the samples were defrosted in air. Analysis of the headspace volatile compounds was carried out in real-time by SIFT-MS. Prior to analysis, three replicate 2.5 mL aliquots of each sample were placed into a sample bag constructed from 50 cm length, 65 mm diameter Nalophan NA (Kalle, UK), which was then filled with purified air and sealed prior to incubation at 37 ^0^C. After equilibrium between the liquid and headspace above it (30 min), the headspace was sampled directly into the SIFT-MS via a heated, calibrated capillary that defines the headspace sample flow rate, as is necessary for absolute quantification of VOCs. The analytical downstream quadrupole mass spectrometer was scanned over the range of mass-to-charge ratio, m/z, using the three reagent ions H_3_O^+^, NO^+^ and O_2_^+^ independently. From the m/z values of the analyte ions and their count rates, and using the kinetics database stored in the instrument library, the concentrations of the identified VOCs were immediately obtained [31, 32].

## Results and discussion

### SIFT-MS analyses

#### Compounds common to biological media

**Fig 1:**
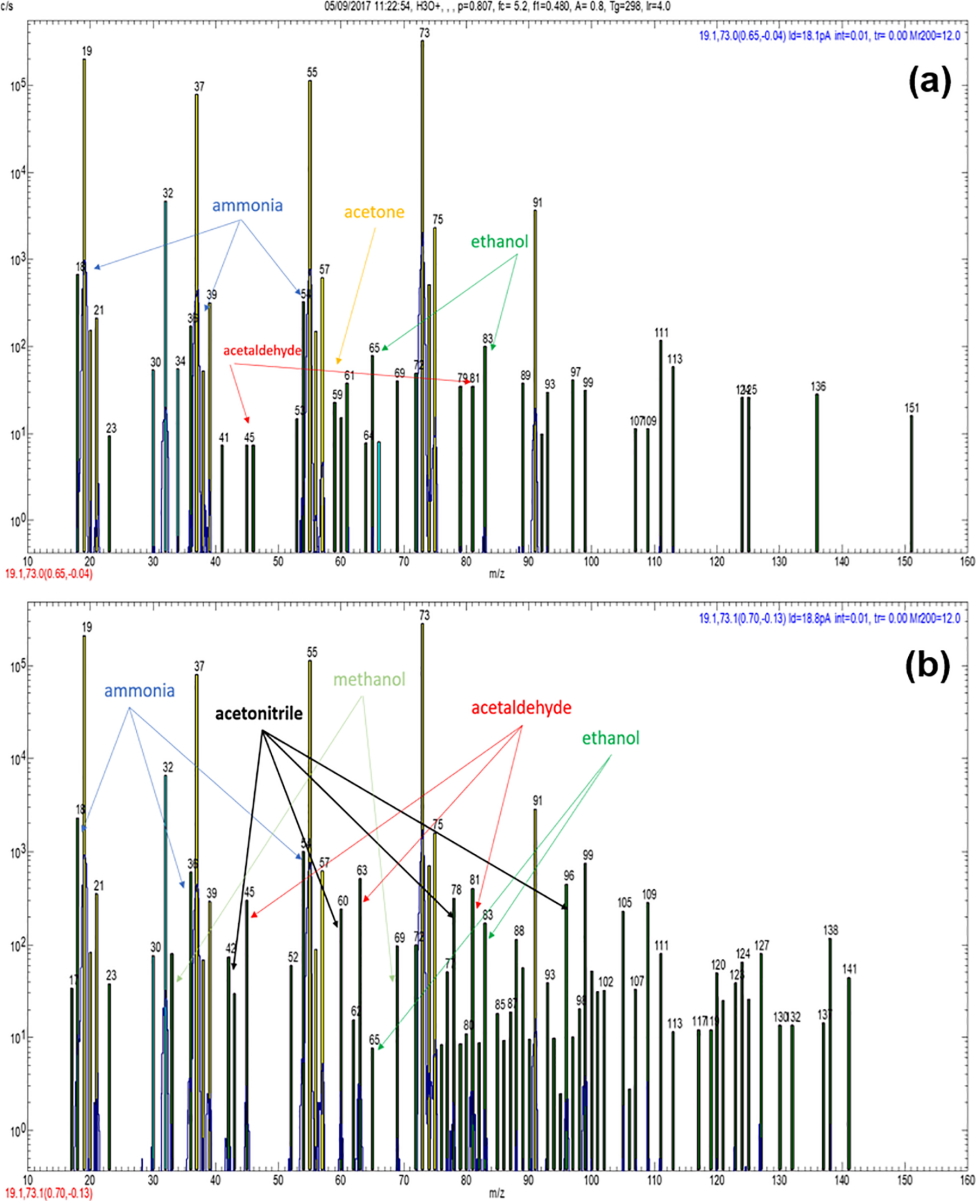
(a) H_3_O^+^ spectrum of control group sample in air. Ions indicating ammonia, acetone, acetaldehyde and ethanol are shown on the spectrum. (b) H_3_O^+^ spectrum of group 4 sample in air. Ions indicating the presence of acetonitrile (42, 60, 78, 96) are indicated. Ammonia, acetaldehyde, ethanol and methanol are also indicated.

**Fig 2:**
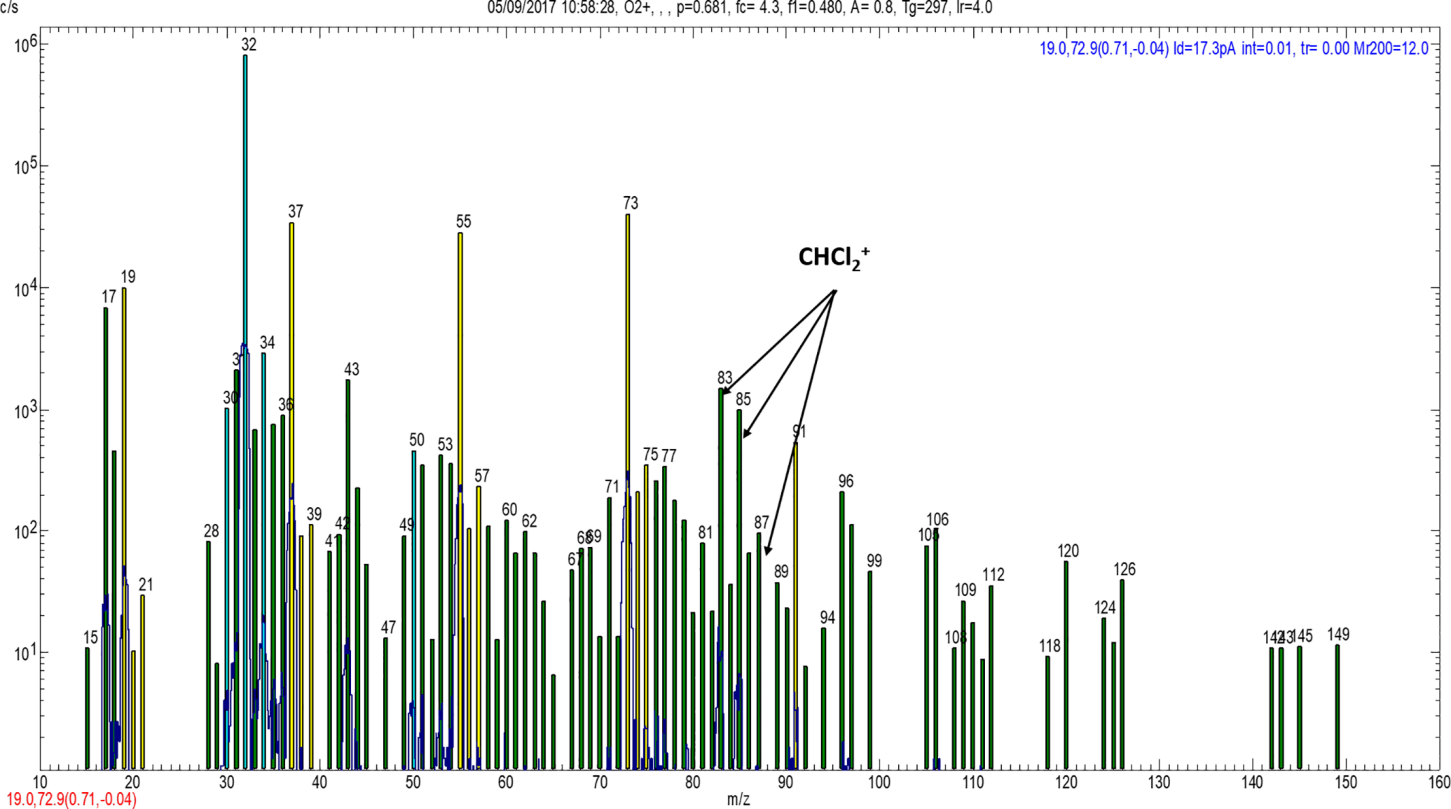
O_2_^+^ spectrum from group 4 in anaerobic conditions showing ions at 83, 85 & 87, and their respective ion counts (83[1485], 85[1342], 87[97]), representing CHCl_2_^+^ derived from the O_2_^+^ reaction with chloroform.

The headspace of all samples contained several common compounds that are often seen in biological media headspace (Table 2) [38]. Sample spectra using the H_3_O^+^ reagent ion for the control group and experimental group 4 are presented in Fig 1, where analyte ions derived from ammonia (NH_3_), acetaldehyde (CH_3_CHO), acetone (CH_3_COCH_3_), methanol (CH_3_OH), ethanol (C_2_H_5_OH) can be seen. In the NO^+^ reagent ion spectra (not reproduced), the characteristic ions for acetic acid (CH_3_COOH) were present. The characteristic ions of NH_3_ were also detected in the O_2_^+^ reagent ion spectra (Fig 2). Details of the ion-molecule reactions by which these compounds are generated, are available in previous papers [31, 32].

**Table 2:** 

Sample	Ammonia Mean [range]	Acetaldehyde Mean [range]	Acetone Mean [range]	Methanol Mean [range]	Ethanol Mean [range]	Acetic acid Mean [range]	Acetonitrile Mean [range]
**Control** **air**	0.654 [0.479–0.869]	0.056 [0.048–0.065]	0.016 [0.014–0.017]	0.089 [n/d-0.178]	0.114 [0.078–0.150]	0.074 [0.040–0.117]	0.009 [n/d-0.017]
**Control** **no air**	0.566 [0.325–0.738]	0.006 [n/d-0.013]	0.069 [0.009–0.128]	0.050 [n/d-0.100]	0.368 [0.103–0.634]	0.098 [0.026–0.210]	0.004 [n/d-0.009]
**Group 1** **air**	0.176 [0.153–0.211]	0.130 [0.128–0.132]	0.038 [0.032–0.043]	0.036 [n/d-0.071]	0.057 [0.006–0.108]	0.119 [0.066–0.149]	0.162 [0.156–0.167]
**Group 1** **no air**	0.234 [0.098–0.497]	0.125 [0.118–0.132]	0.393 [0.294–0.492]	0.019 [n/d-0.039]	0.014 [0.006–0.021]	0.057 [0.035–0.092]	0.299 [0.262–0.335]
**Group 2** **air**	0.409 [0.061–0.936]	0.048 [0.039–0.057]	0.052 [0.040–0.064]	0.091*	0.011 [n/d-0.021]	0.043 [0.030–0.056]	0.174 [0.127–0.221]
**Group 2** **no air**	0.236 [0.180–0.339]	0.045 [n/d-0.089]	0.189 [0.160–0.219]	n/d [n/d-n/d]	0.003 [n/d-0.006]	0.058 [0.014–0.107]	0.272 [0.183–0.359]
**Group 3** **air**	0.152 [0.091–0.192]	0.128 [0.079–0.177]	0.183 [0.167–0.199]	0.045 [n/d-0.090]	0.051 [0.050–0.052]	0.060 [0.053–0.073]	0.400 [0.260–0.540]
**Group 3** **no air**	0.284 [0.120–0.590]	0.048 [0.019–0.076]	0.693 [0.577–0.807]	0.161 [0.040–0.281]	0.041 [0.013–0.069]	0.131 [0.034–0.260]	0.653 [0.581–0.725]
**Group 4** **air**	5.563 [2.270–10.663]	0.825 [0.580–1.070]	0.046 [0.045–0.048]	0.100 [0.069–0.131]	0.093 [0.091–0.095]	0.053 [0.012–0.079]	0.316 [0.226–0.407]
**Group 4** **no air**	6.664 [3.951–10.250]	1.023 [0.815–1.230]	0.046 [0.036–0.057]	0.214 [n/d-0.429]	0.130 [0.111–0.148]	0.102 [0.003–0.227]	0.200 [0.098–0.302]

Mean concentrations and range [minimum-maximum] of 2 or 3 samples (mg L^-1^ by volume) from the analyses by SIFT-MS using H_3_O+ and NO+ reagent ions of some compounds present in the headspace of control group and experimental groups 1–4 in aerobic and anaerobic conditions.

*Only one data point

n/d = non detectable.

The experimental procedures were conducted under both aerobic and anaerobic conditions to justify any alterations in the formation of VOCs and DBPs. From an ecologic perspective, the conditions within infected root canal systems vary at different depths. The coronal third presents high oxygen tension, availability of nutrients from oral cavity and microorganisms can be directly exposed to antimicrobial agents [39]. Oxygen tension and nutrients present a declining tendency from the middle to apical root third. However periapical tissues may offer different sources of nutrition in the apical third and microorganisms are less vulnerable to antimicrobial agents due to anatomical restrictions [39]. After access cavity and during root canal preparation and irrigation, oxygen may progressively diffuse, however the least approachable areas in the apical root third may still be subjected to treatment under oxygen-low or anaerobic conditions [39]. To-date, we have no available data regarding the possible selectivity of NaOCl oxidation capacity under aerobic or anaerobic conditions. The results of this study showed that no significant differences were observed between samples in aerobic and anaerobic conditions, with the exception of acetone, that presented higher concentrations in anaerobic conditions for groups 1, 2 and 3. The reason for this is not clear.

The presence of ammonia in the control group is as a result of the emission from the bacterial population, which is produced from the metabolism of peptides and amino acids, especially via L-aspartate catabolism [40]. Group 4 was significantly different from all the other groups. The NH_3_ concentrations present in the headspace of group 4 samples was several times greater than in the other experimental groups’, both in aerobic and anaerobic conditions. This suggests that NH_3_ is efficiently produced by the reaction of the NaOCl with BSA, since this material is not present in the other samples containing NaOCl (groups 1–3). It is well known that serum albumin and hypochlorite interaction lead to protein injury and degradation [41]. One possible explanation may relate to hydrolysis that protein molecules are undergoing and lead to NH_3_ release [42]. Assuming that chloramine (NH_2_Cl) formation is prominent due to interaction of NaOCl with higher levels of organic matter in group 4, we can suggest that NH_3_ formation may also originate from the decomposition of chloramine in alkaline aqueous conditions according to the reaction: 3NH_2_Cl + 3OH− → NH_3_ + N_2_ + 3Cl− + 3 H_2_O [43]. Apart from NH_3_, a higher level of CH_3_CHO release in group 4 than the control or groups 1–3 was observed, under anaerobic conditions. This implies that a specific reaction of NaOCl with BSA may occur.

#### Nitrogen-containing organic compounds; acetonitrile (CH_3_CN)

Evidence for the presence of amines or amides in the H_3_O^+^ spectrum (Fig 1B) and other spectra (not shown here) was also noticeable. These two compounds were not seen in the control group, but in experimental groups 1–4, aerobically and anaerobically, with the largest signals in groups 2 and 3 (although not substantially larger than groups 1 and 4). The characteristic even numbered ions when using H_3_O^+^ reagent ions usually indicate nitrogen-containing compounds. Thus, the analyte ion at m/z 88 (and its monohydrate at m/z 106) is most probably either C_5_H_13_N (pentylamine) or C_4_H_9_NO (butyramide). The minor peak at m/z 102 (plus its monohydrate m/z 120) is most probably either C_6_H_15_N (dipropylamine) or C_5_H_11_NO (pentanamide). However, there is no doubting the identity of the much more pronounced characteristic analyte ions of acetonitrile (CH_3_CN) in the headspace of all groups containing NaOCl (not present in the to the control group headspace). The reaction of CH_3_CN with H_3_O^+^ reagent ions in humid media almost uniquely produces four analyte ions CH_3_CNH^+^(H_2_O)_0,1,2,3_ with m/z values of 42, 60, 78, 96, which are clearly seen in Fig 1B [44, 45]. It is present in easily quantifiable concentrations by SIFT-MS (Table 2). It is not usually seen in biological samples but it is often seen on human breath in smokers [45].

A major question that needs further investigation is how these volatile nitrogenous compounds are formed and whether their direct analysis by SIFT-MS in the headspace may offer insight into the reactions of NaOCl with proteins and amino acids. One possible explanation for the formation of CH_3_CN is by the reaction of NaOCl with aldehydes and monochloramines, which has previously been investigated using solid phase microextraction (SPME) and GC-MS [46]. Note that acetaldehyde is present in the headspace of our samples. Chloramines form following the degradation of proteins and oxidation of aminoacids by NaOCl [47].

CH_3_CN can be absorbed into the body by inhalation of its vapour and by dermal absorption through the skin and by ingestion. No data are available on its carcinogenic effects in humans; EPA has classified it as non-carcinogenic to humans [48]. Its release when used in root canal irrigation should offer little concern at the relatively low levels that are generated, but an occupational risk assessment has not been performed in dental premises and in particular in endodontic surgeries yet, despite the daily use of large volumes of NaOCl for endodontic treatments. In view of the present discovery, perhaps risk assessment of acetonitrile should be carried out.

#### Production of chloroform (CHCl_3_)

In view of the previous work that has reported the production of organochlorine DBPs when using NaOCl for root canal irrigation [30], the SIFT-MS spectra were carefully inspected for the presence of chlorine-containing analyte ions. Unfortunately, chlorinated organic compounds react only slowly with H_3_O^+^ and NO^+^ and so these reagent ions are not very useful for the analysis of these compounds. However, they usually react rapidly with O_2_^+^ ions [49]. Ions containing one chlorine atom can often be recognised on mass spectra because the peak heights (intensities) of the two isotopologues (separated by two mass-to-charge ratio, m/z, units) will be in the ratio of the naturally occurring abundance ratio of ^35^Cl to ^37^Cl isotopes which is statistically 3:1 (75%:25% peak intensities). Hence, by recognising analyte ions that differ by 2 m/z units that have peak intensity ratios of 3:1, it is possible to identify monochlorinated ions and suggest molecular formulae. For ions containing two chlorine atoms, three isotopologue ions will be formed separated by 2 m/z units with the peak height intensities in the ratio 9:6:1 (56%:38%:6%). Even though the spectra obtained using O_2_^+^ reagent ions are very “busy”, because these energetic reagent ions can fragment analyte neutral molecules and produce multiple mass spectral peaks, as can be seen in Fig 2, analyte ions at m/z values of 83, 85 and 87, with peak intensities close to the statistical ratio expected for an ion containing 2 chlorine atoms, are present in all the spectra obtained for the analysis of the samples containing NaOCl, but not in the control sample spectra. The only candidate for this dichlorinated ion is CHCl_2_^+^. This closed shell ion is known to be the product of the O_2_^+^ reaction with chloroform (CHCl_3_) [49]. Thus, CHCl_3_ is present in the headspace of all the samples containing NaOCl at easily measured concentrations that exceed that of NH_3_ in groups 1–4 (Table 3). To determine whether these findings arose through the reaction of the NaOCl with the organic content (dentine, bacteria and serum) and not the material of the Nalophan sample bag, NaOCl was added to the sample bag to which hydrocarbon free air was added. The bag was incubated for 30 min at 37°C and then analysed by SIFT-MS. No evidence of CHCl_3_, CH_3_CN or any of the other product ions mentioned here were detected, so the findings shown above are not down to reactions of NaOCl with the bag material.

**Table 3:** 

Sample	m/z 83	m/z 85	m/z 87	CHCl_3_ (mg L^-1^)
**Control air**	n/d	n/d	n/d	n/d
**Control no air**	n/d	n/d	n/d	n/d
**Group 1 air**	931 (61)	487 (32)	112 (7)	1.826
**Group 1 no air**	505 (54)	332 (37)	95 (10)	1.038
**Group 2 air**	445 (52)	333 (39)	80 (9)	1.061
**Group 2 no air**	655 (59)	398 (36)	60 (5)	1.292
**Group 3 air**	1092 (48)	935 (41)	245 (11)	2.767
**Group 3 no air**	1372 (59)	783 (34)	150 (6)	2.072
**Group 4 air**	1644 (52)	1342 (43)	152 (5)	3.474
**Group 4 no air**	1485 (58)	983 (38)	97 (4)	2.622
**Mean percentages (%)**	(56)	(38)	(6)	

Ion intensities of ions (count rates) as relative percentages (%) in parentheses at m/z 83, 85 and 87 using O_2_^+^ reagent ions and thus the concentration of chloroform (mg L^-1^ by volume). Note that the mean peak intensity percentages (%) are precisely in line with the statistical predictions (given in the text).

n/d: non detectable.

As mentioned in the Introduction, Varise *et al*. [30] reported the formation of organochlorine compounds, including CHCl_3_, following the 15-min interaction of NaOCl with bovine dentine powder and pulp tissue fragments. In another study, the formation of trihalomethanes and other DBPs was documented using gas chromatography with electron capture detector (GC-ECD), following the 1-h interaction of chlorine (1–3 mgL^-1^) with *E*. *coli* and *Pseudomonas aeruginosa* bacterial cells [50]. The combined findings of bacterial inactivation and DBP formation imply that breaking down bacterial cells provides organic precursors for DBP formation [50]. This evidence strongly supports the pathway for DBP formation from pure bacterial biomaterials [50]. An alternative pathway for the formation of CHCl_3_ may derive from the interaction of NaOCl with acetone according to the historically known haloform reaction: 3NaOCl + C_3_H_6_O → CHCl_3_ + 2NaOH + NaOCOCH_3_ [51].

The production of CHCl_3_, a trihalomethane, may have implications in dentistry [10]. It has been shown to be carcinogenic in animals after oral exposure, resulting in an increase in kidney and liver tumours [52]. The United States Environmental Protection Agency (USEPA) has classified chloroform as a probable human carcinogen [5]. The World Health Organisation (WHO) has concluded that the weight of evidence suggests that chloroform does not have direct genotoxic potential [53], but the European Agency for Safety and Health at Work has established indicative occupational skin exposure limit values for CHCl_3_ at 10 mg (m^3^)^-1^ or 2 mg L^-1^ per 8 working hours [54]. CHCl_3_ is still used in endodontics as a solvent of root canal sealers and gutta-percha in cases of root canal retreatment [55] as well as for the customisation of master gutta-percha cone during root canal obturation [56]. Despite the potentially serious concerns regarding its safety due to dental personnel inhalation exposure risks [57], risk assessment studies report that CHCl_3_ has no negative health effects under careful and controlled use [58, 59]. The results of this study showed that CHCl_3_ formation is evident with all combined components in our experimental group samples and further risk assessments are essential in dentistry.

### Potential complications in endodontic practice and future directives

The results of this study offer an overview of the chemical interactions within chlorinated aliquots of endodontic origin. The detection of VOCs and DBPs and their relevant concentrations should be interpreted with caution as they do not entirely represent conditions of endodontic practice. However, they do indicate that additional chemical compounds that are encountered when NaOCl is used as main root canal irrigant. So, the outcomes of this study cannot be directly extrapolated in clinical conditions, but the formation of DBPs and VOCs implies that the risks and drawbacks from the use of NaOCl in dental clinical procedures require critical review and further appraisal with respect to human and environmental exposure.

Patients and dental staff may accidentally inhale the volatile phase of DBPs formed during the contact of NaOCl with organic substrates in chemo-mechanical and irrigation procedures, representing a potential threat to human health [52]. There is also a risk of inadvertent extrusion of toxic chlorinated compounds into the periapical space and blood circulation during root canal preparation [60]. In addition, the aspiration of chlorinated aliquots with dental unit surgical suction during root canal irrigation may contribute to the accumulation of DBPs as liquid suspensions in sewers and waste-water distribution systems in dental surgeries or hospital premises.

Further studies are required for the examination of the cumulative effects on dental staff and the degree of patient exposure through accidental inhalation of the volatile phase of DBPs during endodontic treatment procedures, under conditions of good practice.

## Conclusions

SIFT-MS is shown to be an effective technique for the real-time analysis of volatile compounds released by the reaction of NaOCl and representative components of an infected root canal system. Within the limitations of this *ex vivo* study and in the rejection of the null hypothesis, the chemical interaction of NaOCl 2.5% with dentine powder, bacteria, bovine serum albumin and their combination resulted in the formation of toxic DBPs and VOCs under both aerobic and anaerobic conditions.
